# In silico characterization and differential expression pattern analysis of conserved HMG CoA reductase domain isolated from *Aconitum balfourii* Stapf

**DOI:** 10.1007/s13205-016-0405-y

**Published:** 2016-03-07

**Authors:** Eti Sharma, Saurabh Pandey, A. K. Gaur

**Affiliations:** 1 Department of Molecular Biology and Genetic Engineering, College of Basic Sciences and Humanities, G. B. Pant University of Agriculture and Technology, Pantnagar, 263145 Uttarakhand India; 2 Plant Molecular Biology Lab, The International Centre for Genetic Engineering and Biotechnology ICGEB, New Delhi, India

**Keywords:** Aconitine, Mevalonate pathway, 3-Hydroxy-3-methyl glutaryl-CoA coenzyme A reductase, HMGR, Expression profiling

## Abstract

The 3-hydroxy-3-methyl glutaryl CoA reductase (HMGR) is the key enzyme of mevalonate pathway in plants. A partial genomic DNA fragment encoding HMGR conserved domain (denoted as AbHMGR) is isolated from *Aconitum balfourii* Stapf. It comprises 871 bp encoding 290 amino acids. In silico analysis reveals that it had extensive similarities to other plant HMGR gene. Domain analysis of AbHMGR showed two highly conserved NADPH and HMG CoA domains. Docking study predicted inhibitor, substrate and cofactor binding sites in the protein. Expression analysis revealed that AbHMGR is similarly expressed in all tested tissues with differential pattern. The highest expression was found in leaf tissue. However, fold expression in root and shoot tissue was almost similar. Enzyme activity of HMGR was found to be much higher in leaf tissue as compared to other tissues. The highest aconitine content (0.015 %) was obtained in root tissues. Our data laid a foundation for further investigation of HMGR role in *Aconitum balfourii.*

**Electronic supplementary material:**

The online version of this article (doi:10.1007/s13205-016-0405-y) contains supplementary material, which is available to authorized users.

## Introduction

*Aconitum balfourii (A. balfourii)* Stapf is one of the endangered herbs of the genus *Aconitum*. The main medicinal material in roots of *A. balfourii* Stapf is a diterpenoid alkaloid (Sultankhodzhaev and Nishnov 1995). The value of aconitine as a medicine has been recognized in modern times, and it now ranks as one of the most useful drugs, particularly in homeopathy, Ayurveda and Unani systems of medicine (Kirtikar and Basu 1965). It cures several ailments like rheumatism, arthritis, gout, neuralgia, sciatica, migraine and cancer. In homeopathy, aconite is used to dispel fear, anxiety and stress (Fleming 2000; Garmanchouk et al. 2005).

Aconitine(s) are the part of diterpenoid alkaloids which originate from a terpenoid backbone (Cherney and Baran 2011). All terpenoids in plants are synthesized via central intermediate isopentyl pyrophosphate (IPP). IPP formation follows two distinct routes, the classical mevalonate pathway (MVA) which is effective in cytosol and another one is 2-C-methyl-d-erythritol 4-phosphate (MEP) in plastids (McGarvey and Croteau 1995). In MVA pathway, 3-hydroxy-3-methyl glutaryl-CoA reductase (HMGR) is a key enzyme. It catalyzes the first committed step in which three molecules of acetyl Co A condense successively to form 3-hydroxy-3-methyl glutaryl-CoA (HMG CoA). The HMG-CoA is then reduced to yield mevalonic acid in an NADPH-dependent double reduction. This step is catalyzed by mevalonate:NADP oxido reductase, CoA acylating; 3-hydroxy-3-methylglutaryl coenzyme A reductase (HMGR; EC 1.1.1.34) (Rogers et al. 1983). Evidence for the contribution of HMGR as the rate-limiting enzyme in isoprenoid biosynthesis has come from several investigators (Chappell and Nable 1987; Narita and Gruissem 1989). The major sub-cellular location of the enzyme appears to be the endoplasmic reticulum (ER) membrane. HMGR activity has also been reported to be associated with mitochondria and plastids (Laule et al. 2003).

In the last few years, *A. balfourii* Stapf faces severe threat due to overexploitation. For saving the plant from extinction it is a necessity now to apply some biotechnological approaches other than tissue culture. Gene mining of rate-limiting enzymes of aconitine biosynthesis pathway and their studies is one of the strategies which needs to be focused. Fishing out the probable rate-limiting gene(s) and in silico characterize them is the beginning step. Until now, no information is available regarding cloning and characterization of HMGR full length or partial gene sequence from any species of genus *Aconitum*. Though HMGR genes have been isolated from many other plant species such as *Camptotheca acuminata* (Maldonado et al. 1997 *), Catharanthus roseus* (Maldonado-Mendoza et al. 1992), *Melon* (Kato-Emori et al. 2001), and *Taxus media* (Liao et al. 2004). In the view of above, we successfully attempted the isolation of HMG CoA reductase gene from *A. balfourii* Stapf. The gene is designated as AbHMGR and its characterization was done with the help of bioinformatics tools. Expression pattern analysis and HMGR enzyme activities and aconitine content analysis at tissue level were also performed.

## Materials and methods

### Plant materials

*Aconitum balfourii* was procured from Tungnath region (3300 m) of Uttarakhand (India). In vitro fully differentiated cultures of *A. balfourii* were established (Sharma et al. 2012) in tissue culture and these cultures were used for various analyses.

### Screening of HMGR sequences and cloning of AbHMGR

We retrieved the HMG sequences from more closely related families to Ranunculaceae (Grund et al. 1981) from NCBI database (http://www.ncbi.nlm.nih.gov). All the sequences were aligned by using Mega 4.0 software (Tamura et al. 2007). Primers were designed from obtained conserved regions by Primer3 online tool (Untergasser et al. 2007). The sequence of successful primer was HMGF GGCAACCACTGAAGGATGTT and HMGR ATGTTCTGAGCTGGGTCCTG. Amplification was carried out according to the following temperature profile: 5-min initial denaturation at 95 °C; 35 cycles of 94 °C for 1 min, 55 °C annealing temperature based on the Tm value of the primers for 1 min, 72 °C for 1 min; final extension of 10 min at 72 °C; and final hold at 4 °C. A single approximate 900-bp fragment was amplified which was directly cloned in pGEMT easy vector (Promega, USA) as per the kit instructions. Putative cloned HMG gene was sequenced using M13 universal primer in p-GEMT easy vector.

### In silico analysis

The obtained sequence of AbHMGR was analyzed using several online and offline web services and softwares. Homology search was performed by BLASTX (Altschul et al. 1997). Sequence was translated into protein sequence using Expasy tool (http://ca.expasy.org/tools/dna.htmL). Protein functional analysis was done using INTERPROSCAN version 4.4 (Quevillon et al. 2005). Multiple sequence alignment of HMGR proteins was performed using ClustalW (Thompson et al. 1994) with default parameters. Phylogenetic relationship among other sequences was analyzed by Molecular Evolutionary Genetic Analysis (MEGA) software (version 5.2) software (Tamura et al. 2011) using UPGMA method. Each node was tested using the bootstrap approach by taking 1000 replicates. MEME version 4.8.1 was used for the elucidation of motifs in sequences (Bailey et al. 2009) and the motif widths were constrained to between 6 and 50 residues.

### Docking study

Docking study was also conducted and structure of all chemical compounds, i.e., sodium pyruvate, fosmidomycin, NADH, HMG Co A, mevinolin and NADPH were downloaded from PUBCHEM database. Docking with all ligands and three-dimensional structures of putative genes were performed using AutoDock, an automated docking tools (http://autodock.scripps.edu/). For simulation Discovery studio (http://accelrys.com/products/discovery-studio/) was used. The model of HMGR gene product was generated using Modeller 9.12 by comparative modeling of protein structure prediction. The model was evaluated on the basis of geometrical and stereo-chemical constraints using PROCHECK, ProSA-Web (Wiederstein and Sipple 2007) and verify 3D (Eisenberg et al. 1997). Root mean squared deviation (RMSD) was calculated using MOE. For searching the interacting partner proteins String database was used (http://string-db.org/).

### Expression pattern analysis

Total RNA was extracted from plant tissues using the RNeasy Plant Mini kit (Qiagen, USA) and pre-treated with RNase-Free DNase (Fermentas International, Canada) to eliminate genomic DNA contamination. RNA integrity was analyzed on a 1 % argarose gel. RNA quantity was determined using an nanodrop 2000 °C spectrophotometer (Thermo Scientific, USA). Total RNA was used to synthesize first-strand cDNA by using oligo (dT)_18_ primer with RevertAid H Minus M-MuLV RT (Fermentas International Inc.). Actin gene was also amplified using specific primers ActF GTGCAATGGAACTGGAATGG and ActR AGACGGAGGATAGCGTGAGG (Kai et al. 2006) yielding a single band of 500 bp and it was confirmed by sequencing.

### Quantitative Real-Time PCR

Real-time PCR was performed using QuantiFast SYBR Green PCR kit (Qiagen, USA). The amplified gene sequence of actin from *A. balfourii* was used to design the real-time PCR primers that give 158 bp product size. The primer sequences for internal control actin were: AbactF CTTACAGAAGCACCCTTGAACC and AbactR TCACCAGAATCCAGCACAATAC. The following amplification program was used: 95 °C for 5 min, 40 cycles at 95 °C for 10 s, 60 °C for 30 s, 72 °C for 30 s; 60 °C for 15 s and 95 °C for 15 s. All samples were amplified in triplicate, and the mean value was considered. The relative value obtained for quantitation was expressed at 2^−ΔΔCT^, where ΔCT represents the difference between the CT value of the sample and that of actin (endogenous control) in the same sample, and ΔΔCT is difference between the ΔCT value of a sample and that of its respective control (Livak and Schmittgen 2001).

### HMGR activity

Protein was extracted from leaf, root and shoot tissues using extraction buffer containing 50 mM Tris–HCl, 10 mM of β-mercaptoethanol, 1 % w/v PVP and pH was adjusted to 7.5. The tissues were ground in the extraction buffer (1 g fw/ml) for 5 min with pestle and mortar on ice. The extract was transferred to Eppendorf and was centrifuged at 10,000 rpm for 30 min at 4 °C for obtaining solid free extract. The HMGR activity was determined by the method of (Toroser and Huber 1998) with slight changes. The enzyme extract was added (25 μg protein per ml) to a 50 mM of Tris–HCl buffer (pH 7.0) containing 0.15 mM HMG CoA (Sigma), 0.1 mM NADPH and 4 mM of DTT (1,4-dithiothreitol). NADPH oxidation in the reaction solution was monitored at 25 °C by the decreasing absorbance at 340 nm, against the solution free HMG CoA as blank. One HMGR enzyme unit is equivalent to the oxidation of 1 µmol of NADPH per minute.

### HPLC analysis

Aconitine alkaloid was extracted from the dried leaf, shoot and root tissues by using ammoniacal ether–methanol method with the help of high-performance liquid chromatography(HPLC) (Hikino et al. 1983). All the samples were filter sterilized using 0.45 µ nylon filter before injecting in HPLC. The chromatographic separation was carried out using reverse phase C_18_ column with isocratic mobile phase methanol and water in 60:40, respectively. The identification of aconitine was based on the retention time and comparison of the authentic standard purchased from Chromadex. Quantification analysis was repeated for three replicates of each tissue in parallel, and the means and standard deviations were calculated.

### Statistical analysis

All the experiments of enzyme assays, expression analysis and alkaloid estimation at tissue level were performed in triplicates. Mean values of various treatments were subjected to two-way analysis of variance (ANOVA). In gene expression analysis, three independent determinations for each parameter were recorded and mean ± SE values were calculated for statistical analysis. CRD were used for analyzing gel data and real-time data. *Z* score analysis was performed to find out correlation among gene expression, enzyme activity and aconitine content at tissue level.

## Results and discussion

### Cloning of AbHMGR partial gene

A 871 bp partial genomic fragment was isolated from *A. balfourii* Stapf. The vector sequence was wiped off with the help of VecScreen online tool from putative sequenced product. The putative sequence was subjected to BLAST search in GenBank database (http://www.ncbi.nih.gov) and homology search confirmed the identity of HMGR sequence. It is denoted as AbHMGR (accession no. KC514134.1) which is 871 bp and encodes 290 amino acids.

### Multiple sequence alignment and phylogenetic analysis

Multiple sequence alignment of AbHMGR and 21 other plants HMGR sequences (Online Resource 1) was carried out for comparative analysis and it showed the wide-ranging similarities in nucleotide sequence of AbHMGR and other plants HMGR genes. Translated protein sequences were subjected to protein blast to reveal the similarity at protein level with other existing HMGR sequences. Its deduced amino acid sequence showed extensive similarity to HMGR gene from other flowering plants, e.g., *Vitis vinifera* (CBI40773.3, 90 % identity), *Panax quinquefolius (*ACV65036.1, 89 % identity), *Ricinus communis* (XP_002510732.1, 87 % identity) and *Morus alba* (AAD03789.1, 90 % identity). Multiple sequence alignment exposed high degree of sequence conservation in HMGR of the *Aconitum* and other plants. Besides this BLAST search revealed it belongs to HMG CoA reductase superfamily.

**Fig. 1:**
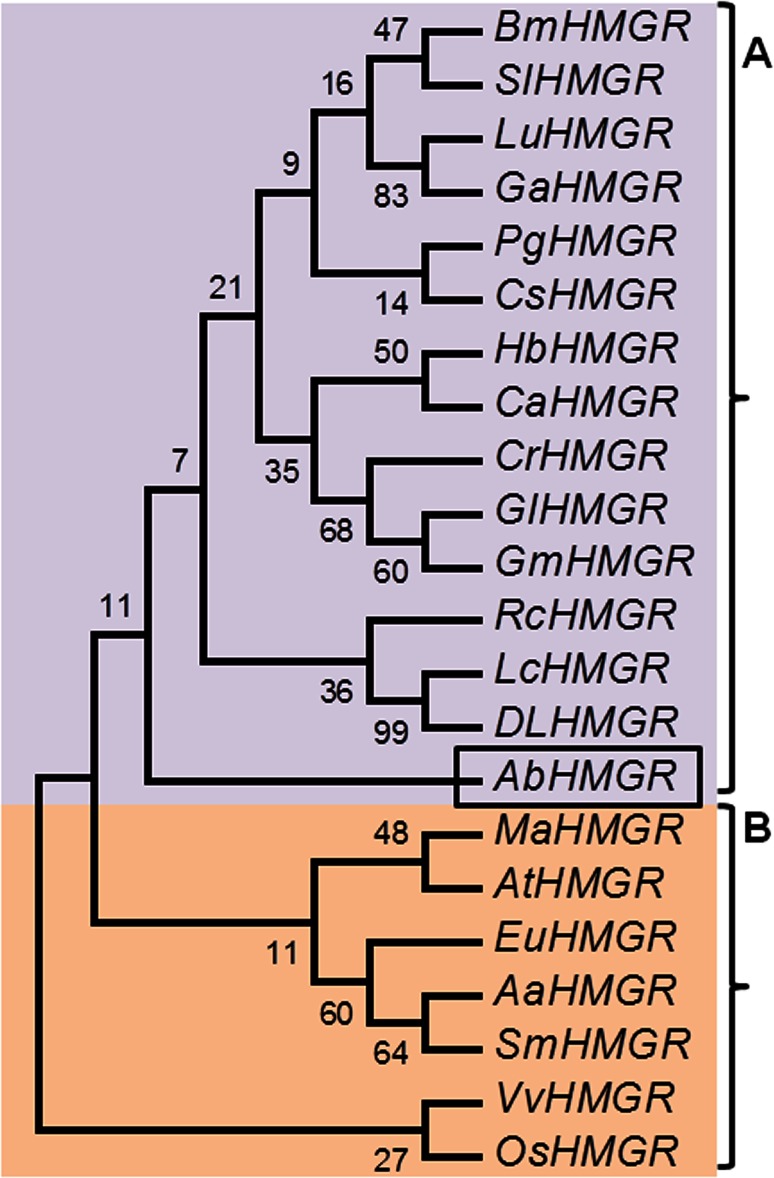
Phylogenetic tree of AbHMGR proteins from different plant species. Sequence analysis was performed using ClustalW and the UPGMA method was applied to create trees. AbHMGR (AGK24692.1) and related proteins from *Panax quinquefolius (*ACV65036.1), *Ricinus communis* (XP_002510732.1), *Solanum lycopersicum* (AL16927.1), *Morus alba* (AAD03789.1), *Litchi chinensis* (ABF56518.2), *Dimocarpus longan* (AET72044.1), *Eucommia ulmoides* (AV54051.1), *Coffea arabica* (ADR51242.1), *Gentiana macrophylla* (AFN89599.1), *Linum usitatissimum* (ACN38874.1), *Hevea brasiliensis* (BAF98280.1), *Bacopa monnieri* (ADX01170.1), *Artemisia annua* (AAA68966.1), *Catharanthus roseus* (AAT52222.1), *Salvia miltiorrhiza* (ACD37361.1), *Camellia sinensis* (AHB64333.1), *Gentiana lutea* (BAE92730.1), *Vitis vinifera* (CBI40773.3), *Gossypium arboreum* (KHG04251.1), *Arabidopsis thaliana* (NP_177775.2), *Oryza sativa* Japonica group (Os08g0512700)

A phylogenetic tree was constructed and phylogenetic relationship of AbHMGR protein with other taxa is shown in (Fig. 1). According to phylogenetic analysis, four clusters are emerging from the tree. AbHMGR emerges as a separate branch from the tree showed its distant relationship to other plant HMGR considered for analysis. It showed higher similarity to other plants class 1 HMGR so it was considered in HMGR1 category.

### Motif prediction

**Fig. 2:**
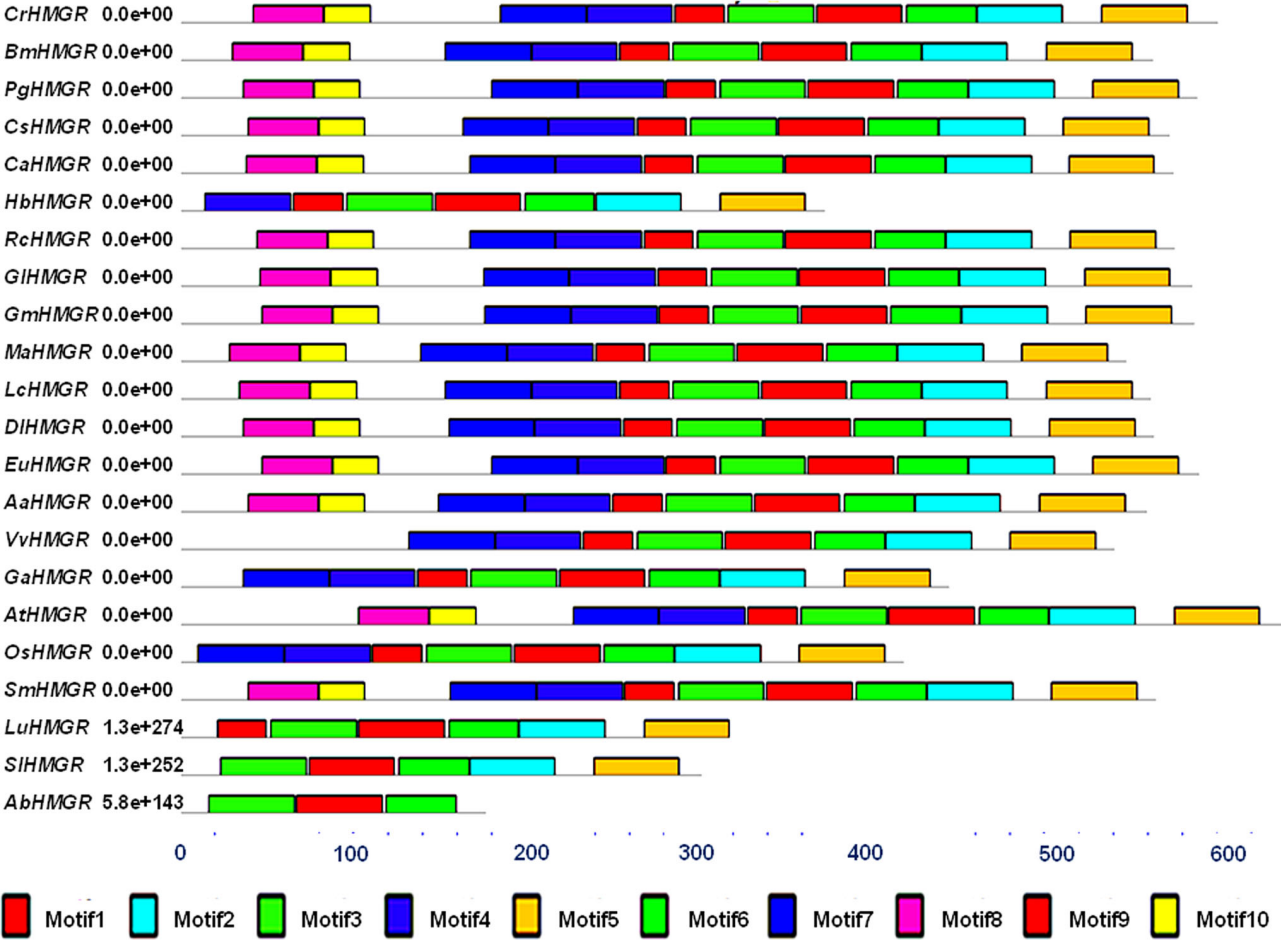
Distribution of motifs among 22 sequences subjected to MEME

A total of ten motifs labeled as 1–10 were observed in 22 sequences when subjected to MEME. The distribution of these motifs among 22 HMGR sequences with accession number is shown in Fig. 2. Conserved domains were also predicted with the help of INTERPROSCAN. AbHMGR contained NADPH and HMG CoA-binding domains. The motif analysis revealed that AbHMGR contain NADPH-binding domain (DAMGMNM) and HMG CoA-binding domain (TTVASLVE). Plant HMGR comprised two HMG CoA-binding motifs (EMPIGYVQIP and TTEGCLVA) and two NADPH-binding motifs (DAMGMNM and GTVGGGT) (Kato-Emori et al. 2001; Liao et al. 2004; Jiang et al. 2006; Shen et al. 2006). The sequence analysis revealed that the functional NADPH-binding domain of AbHMGR was similar to those of other plant HMGRs, but HMG CoA-binding domain showed variations with other plants HMGR conserved motif.

### Docking study

**Fig. 3:**
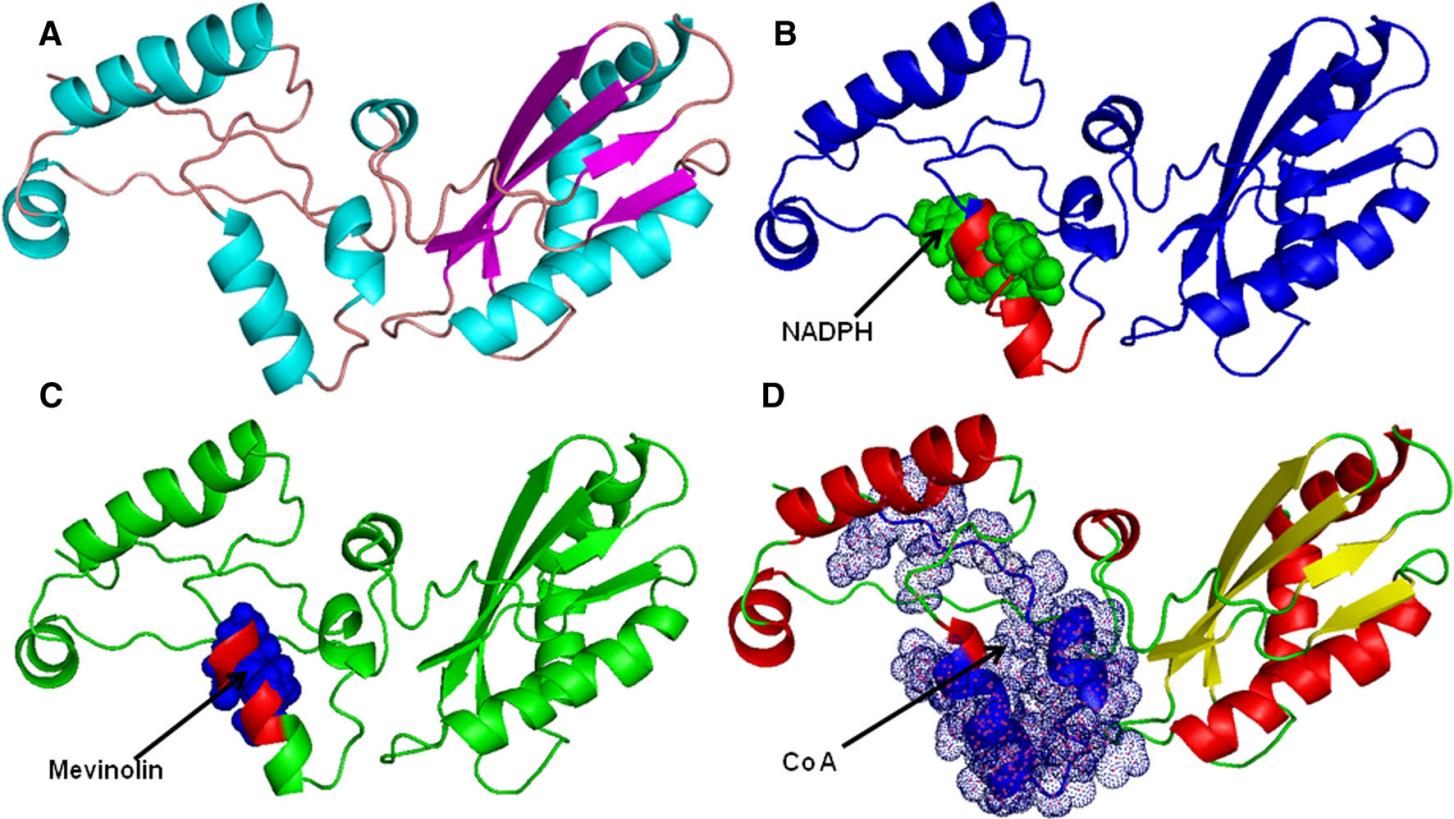
Docking study of AbHMGR protein of *A. balfourii* Stapf. **a** The modeled overall 3D structure of AbHMGR, **b** AbHMGR docked with NADP(H), **b** AbHMGR docked with mevinolin, **c** AbHMGR docked with CoA. The *bindings* of all three ligands are indicated

Docking study was performed with all 3 ligands, i.e., HMG CoA substrate for AbHMGR, NADP(H) as cofactor and mevinolin as specific inhibitor of this enzyme. It was observed that the residues bind with their specific sites perfectly. The small substrate molecule NADP(H) was docked inside the cavity of HMG protein. We checked the minimum free energy of the receptor protein structure by taking into account the flexibility of the side chains before and after binding of the ligand in binding site residues using PROCHECK. Sybyl generated 10 best docked conformations of the substrate based on various scoring algorithms such as crash, polar, D score, PMF-score, G score, ChemSCO and C Score. The best docked conformation was selected based on consensus score for molecular interaction analysis. The strength of individual scoring functions was combined to produce a consensus that is more robust and accurate than any single function for evaluating ligand receptor interactions. The residues which involve in docking are presented in Table 1. The binding of ligands with protein AbHMGR is presented in Fig. 3.

**Table 1:** Interaction energy and residues involved in docking of AbHMGR with different ligands

Substrate	Interaction energy (kcal/mol)	No. of residues	Residues involved in docking
HMG Co A	−28.23	23	Val120, Cys121, Glu122, Ala123, Asn145, Ala166, Ile169, Val170, Ser171, Ala172, Val173, Phe174, Ile175, Gln179, Asn180, Arg181, Ile182, Ala184, Ala188, Ser191, Met192, Arg193, Arg194
NADPH	−28.49	19	Val119, Cys121, Glu122, Ala123, Ala166, Ser167, Asn168, ILE169, Val170, Ser171, Ala172, Val173, Phe174, Ile175, Thr177, Arg181, Ile182, Ala184, Ala188
Inhibitors			
Mevinolin acid	−32.15	16	Cys121, Asn163, His165, Ala166, Ser167, Asn168, Ile169, Val170, Ser171, Ala172, Val173, Phe174, Thr177, Ala184, Ala188, Met192

To find out the interacting partner of AbHMGR, it was subjected to String Database. It showed the several other proteins interact with AbHMGR protein (Online Resource 2). AbHMGR interact with DXR (1-deoxy-d-xylulose 5-phosphate reductoisomerase), MK (mevalonate kinase; encodes a protein with mevalonate kinase activity), IPP1(isopentenyl-diphosphate delta-isomerase), CLA1 (cloroplastos alterados 1; 1-deoxy-d-xylulose-5-phosphate synthase), MVA1 (acetyl-CoA C-acetyltransferase/hydroxymethylglutaryl-CoA synthase), MVD1 (diphosphomevalonate decarboxylase), HMG2 (hydroxymethylglutaryl-CoA reductase 2), SQS1 (farnesyl-diphosphate farnesyltransferase; encodes squalene synthase), NIA1 (nitrate reductase) and NIA2 isoform of nitrate reductase. DXR (1-deoxy-d-xylulose 5-phosphate reductoisomerase), MK (mevalonate kinase; encodes a protein with mevalonate kinase activity), IPP1 (isopentenyl-diphosphate delta-isomerase), CLA1 (cloroplastos alterados 1; 1-deoxy-d-xylulose-5-phosphate synthase), MVA1 (acetyl-CoA C-acetyltransferase/hydroxymethylglutaryl-CoA synthase), MVD1 (diphosphomevalonate decarboxylase), HMG2 (hydroxymethylglutaryl-CoA reductase 2), SQS1 (farnesyl-diphosphate farnesyltransferase; encodes squalene synthase), NIA1 (nitrate reductase) and NIA2 isoform of nitrate reductase.

### Expression analysis

**Fig. 4:**
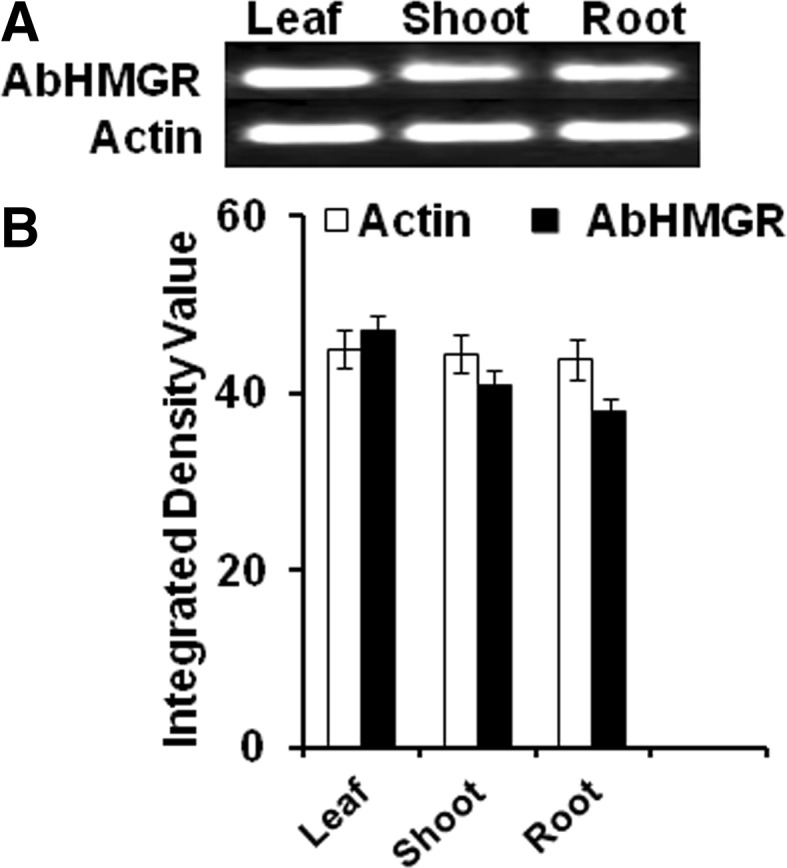
**a** Expression pattern of AbHMGR in different tissues of *A. balfourii* Stapf. Total RNA was isolated from leaves, roots and stems, respectively, was subjected to real-time PCR amplification (*upper panel*). Actin gene was used as the control to show the normalization of the templates in PCR reactions (*lower panel*). **b** Relative expression of AbHMGR in different tissues using real-time PCR. Final data are represented in graphical form. Three independent determinations for each parameter were recorded and mean ± SE values were calculated. CRD were used for analyzing gel data and real-time data

The result showed that AbHMGR expression was detected in all tested tissues which imply that AbHMGR may be a constitutively expressing gene (Fig. 4a).The highest expression in leaves, followed by in shoot and root tissues as in other plants *Corylus avellana* (Wang et al. 2007), American ginseng (Wu et al. 2012) and in *Taraxacum brevicorniculatum* (Deenen et al. 2012). However, fold expression in root and shoot tissues were almost similar. The highest expression (6.02) was found in leaf tissue, followed by shoot (4.72) and root (1) fold (Fig. 4b). Tissue-specific enzyme activities were determined in different tissues viz leaf, shoot and root of *A. balfourii* Stapf. In leaf tissue HMGR activity was found to be much higher (1208 × 10^−15^ IU mg^−1^ protein) as compared to other tissues. Root tissue contained fairly high activity (512 × 10^−15^ IU mg^−1^ protein) and lowest activity was in shoot (133.2 × 10^−15^ IU mg^−1^ protein). Enzyme activities were found to be different at tissue level. Aconitine content was analyzed in different tissues by HPLC. The aconitine content was present in all tissues used for extraction. Maximum content (0.015 %) was in root tissues, followed by stem (0.0028 %) and leaves (0.0011 %).

### Z score analysis

**Fig. 5:**
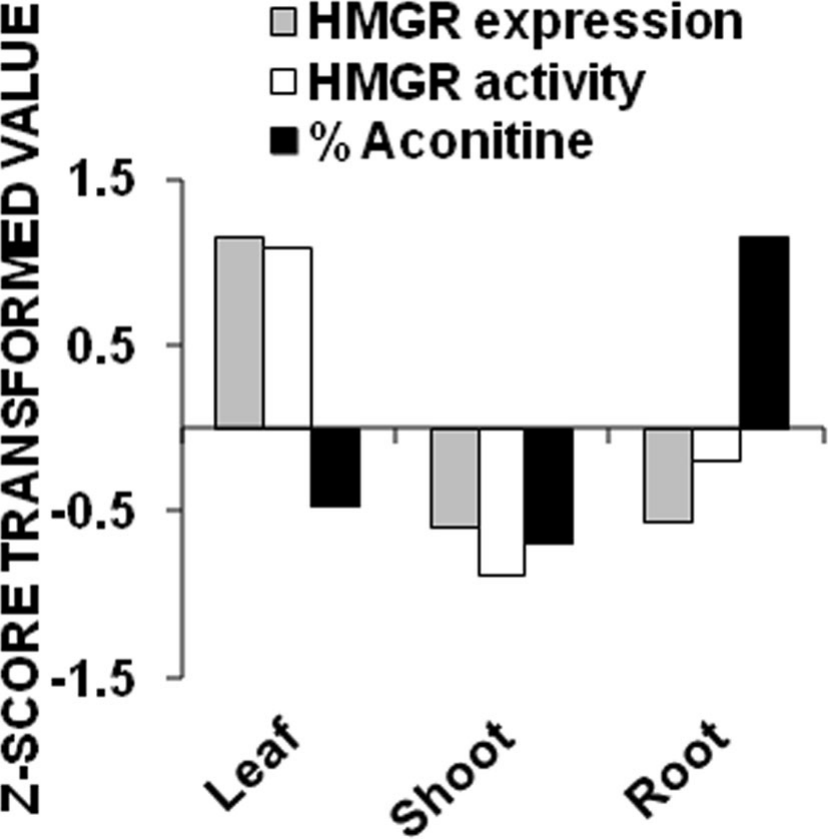
Graphical form of Z score transformation analysis for correlation study among AbHMGR gene expression, enzyme activity and percent aconitine in different tissues of *A. balfourii* Stapf

Z score analysis was conducted to find out the correlation among gene expression, enzyme activity and aconitine content. A negative correlation was obtained as the expression and activity of AbHMGR was found to be highest in leaf tissue, whereas aconitine percentage order was exactly reverse. The highest content was obtained in root tissues followed by leaf and shoot tissues (Fig. 5).

Plant HMGR has a key regulatory role in the MVA pathway, critical not only for normal plant development, but also for the adaptation to demanding environmental conditions. In our study, aconitine content is negatively correlated with gene expression and enzyme activities data at tissue level. It can be explained as, in the *Arabidopsis thaliana*, two genes HMG1 and HMG2 encodes three HMGR isoforms; HMGR1S, HMGR1L and HMGR2. The HMGR1S transcript level is found in all tissues but at fairly high level during the first stage of development of inflorescence. In contrast, HMGR1L and HMGR2 transcripts are only detected in seedlings, roots and inflorescence and are about 10 times less abundant than the HMGR1S mRNA. These observations suggest a housekeeping role for HMGR1S and a more specialized function for HMGR1L and HMGR2 (Enjuto et al. 1995). We got the expression and activity of HMGR in all tested tissues and it is more likely that we isolated HMGR1S fragment. These arguments would explain why we got the negative correlation among aconitine content, gene expression and enzyme activities at tissue level. However, several experiments need to be performed to prove it.

Overall, we have successfully isolated and characterized in silico the functional partial gene encoding HMGR from *A. balfourii* Stapf for the first time. HMGR has been considered as key enzyme of MVA pathway in plants; it is an interesting step to gain knowledge about its structure and function. It might be helpful to further investigate the role of HMGR in aconitine synthesis and also opens the way for applying genetic engineering approaches for enhancement of its medicinal qualities.

## Electronic supplementary material

Below is the link to the electronic supplementary material.

Supp. 1: Multiple sequence alignment of deduced amino acid sequences AbHMGR (AGK24692.1) and related proteins from *Panax quinquefolius (*ACV65036.1),*Ricinus communis* (XP_002510732.1), *Solanum lycopersicum*(AL16927.1), *Morus alba* (AAD03789.1), *Litchi chinensis* (ABF56518.2), *Dimocarpus longan* (AET72044.1), *Eucommia ulmoides* (AV54051.1), *Coffea Arabica* (ADR51242.1), *Gentiana macrophylla* (AFN89599.1), *Linum usitatissimum* (ACN38874.1), *Hevea brasiliensis* (BAF98280.1), *Bacopa monnieri* (ADX01170.1), *Artemisia annua* (AAA68966.1), *Catharanthus roseus* (AAT52222.1), *Salvia miltiorrhiza* (ACD37361.1), *Camellia sinensis* (AHB64333.1), *Gentiana lutea* (BAE92730.1), *Vitis vinifera* (CBI40773.3), *Gossypium arboreum* (KHG04251.1), *Arabidopsis thaliana* (NP_177775.2), *Oryza sativa* Japonica group (Os08g0512700) (TIFF 11273 kb)

Supp.2: Protein protein interaction study of AbHMGR using string database showing interacting partners of AbHMGR (JPEG 31 kb)

## Abbreviations

IPP: Isopentyl pyrophosphate
HMGR: 3-Hydroxy-3-methyl glutaryl reductase
MVA: Mevalonate pathway
MEP: 2-C-methyl-d-erythritol 4-phosphate
