# Supplementary figures and images for: In silico characterization and differential expression pattern analysis of conserved HMG CoA reductase domain isolated from Aconitum balfourii Stapf
Source: 3 Biotech. 2016 Mar 7;6(1):89. doi: 10.1007/s13205-016-0405-y (PMC4781813; doi:10.1007/s13205-016-0405-y)

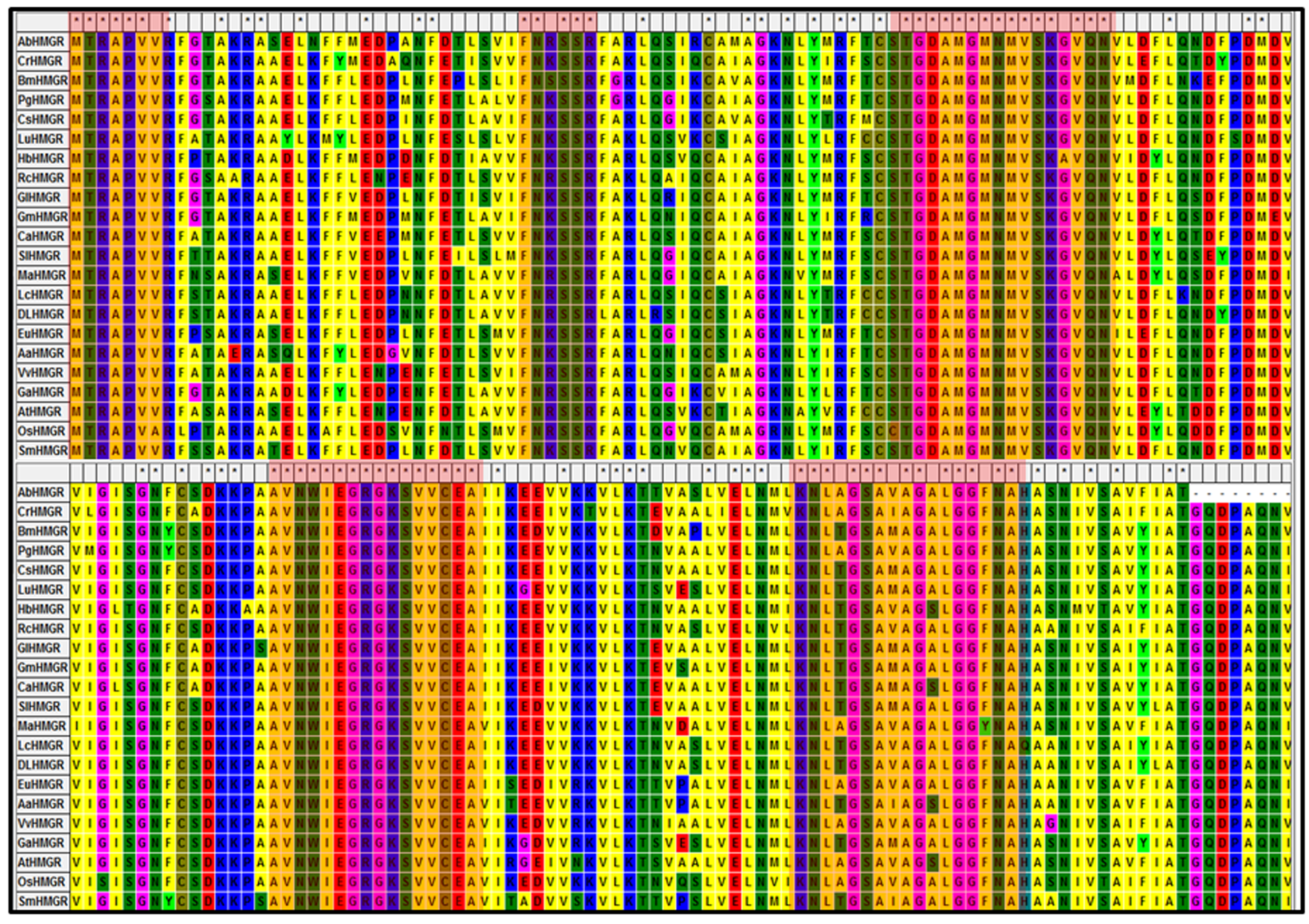

Supplement: Supplementary file 1 — Supp. 1: Multiple sequence alignment of deduced amino acid sequences AbHMGR (AGK24692.1) and related proteins from Panax quinquefolius (ACV65036.1),Ricinus communis (XP_002510732.1), Solanum lycopersicum(AL16927.1), Morus alba (AAD03789.1), Litchi chinensis (ABF56518.2), Dimocarpus longan (AET72044.1), Eucommia ulmoides (AV54051.1), Coffea Arabica (ADR51242.1), Gentiana macrophylla (AFN89599.1), Linum usitatissimum (ACN38874.1), Hevea brasiliensis (BAF98280.1), Bacopa monnieri (ADX01170.1), Artemisia annua (AAA68966.1), Catharanthus roseus (AAT52222.1), Salvia miltiorrhiza (ACD37361.1), Camellia sinensis (AHB64333.1), Gentiana lutea (BAE92730.1), Vitis vinifera (CBI40773.3), Gossypium arboreum (KHG04251.1), Arabidopsis thaliana (NP_177775.2), Oryza sativa Japonica group (Os08g0512700) (TIFF 11273 kb) [file 13205_2016_405_MOESM1_ESM.tif]

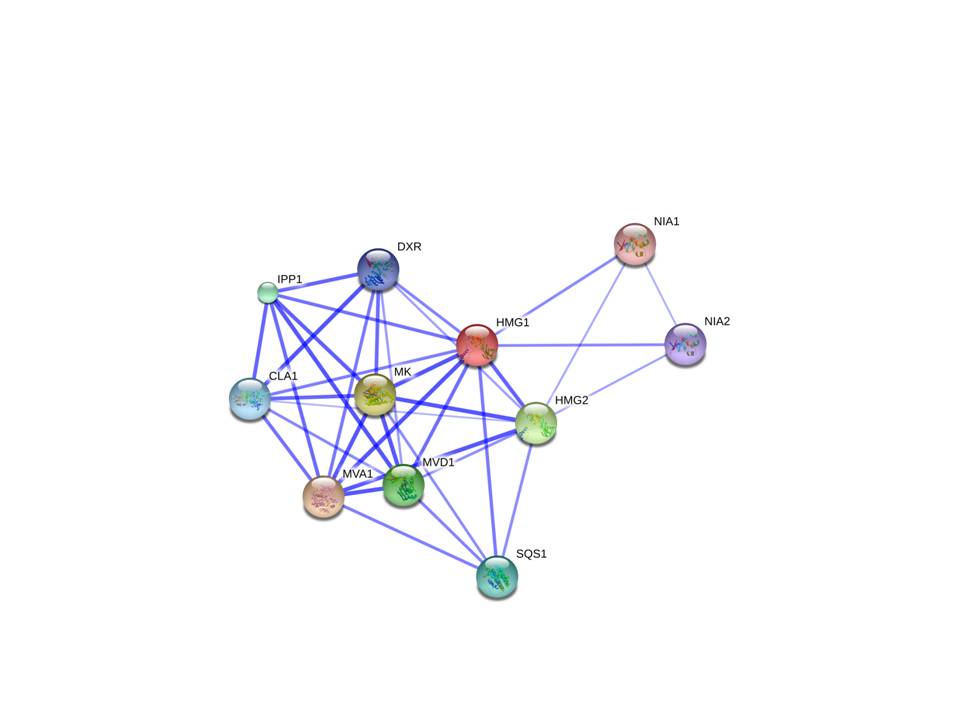

Supplement: Supplementary file 2 — Supp.2: Protein protein interaction study of AbHMGR using string database showing interacting partners of AbHMGR (JPEG 31 kb) [file 13205_2016_405_MOESM2_ESM.jpg]
